# Human resource management and the COVID-19 crisis: implications, challenges, opportunities, and future organizational directions

**DOI:** 10.1017/jmo.2021.15

**Published:** 2021-04-19

**Authors:** Salima Hamouche

**Affiliations:** Faculty of Management, Canadian University Dubai, Dubai, UAE

**Keywords:** COVID-19, crisis, human resource management (HRM), remote work, work from home

## Abstract

The COVID-19 has grandly shaken all organizations, creating a complex and challenging environment for managers and human resource management (HRM) practitioners, who need to find ingenious solutions to ensure the continuity of their companies and to help their employees to cope with this extraordinary crisis. Studies addressing the impact of this crisis on HRM are sparse. This paper is a general literature review, which aims at broadening the scope of management research, by exploring the impact of the COVID-19 on HRM. It identifies the main challenges and opportunities that have arisen from this new pandemic and it offers insights for managers and HRM practitioners into possible future organizational directions that might arise from these opportunities.

## Introduction

COVID-19 is an unprecedented health crisis that has strongly shaken the whole world, plunging it into great fear and uncertainty. It has heavily impacted economies, societies, employees, and organizations. This crisis has started first in the city of Wuhan (China), which has witnessed in December 2019 the outbreak of severe acute respiratory syndrome coronavirus 2 (SARS-CoV-2) that has known a fast spread propelling its status to a global pandemic on March 11, 2020, by the World Health Organization (WHO, 2020b).

Given the rapid spread of the COVID-19 virus, these countries have implemented several non-pharmaceutical measures intended to reduce its spread, such as social distancing. Lockdown measures have been imposed; people were quarantined; schools, universities, nonessential businesses, and non-governmental organizations have been temporarily closed; travels were restricted; flights were canceled; and mass public gathering as well as social events have been prohibited (Brodeur, Gray, Islam, & Bhuiyan, 2020; Gourinchas, 2020).

Coupled with these measures, the COVID-19 outbreak had led to a significant slowdown in the world economic activities (Brodeur et al., 2020; Gourinchas, 2020), triggering furloughs and layoffs (World Economic Forum, 2020), that led to the increase in the unemployment rate in many countries. The ‘Current G7 jobless totals vary widely, from 30 million in the United States to 1.76 million in Japan’ (Kretchmer, 2020). According to Gourinchas (2020), COVID-19 has generated a situation where in a short period 50 percent or more of the workforce might not be able to work.

Trying to recover from this economic shock, companies have started reopening (Major & Machin, 2020), in the mid of this ongoing pandemic, under extraordinary rules and a new functioning (e.g., physical distancing in the workplace) (Shaw, Main, Findley, Collie, Kristman, & Gross, 2020) that no one can predict when it will end. Therefore, this pandemic has obviously led to the emergence of a complex and challenging environment for managers and human resource management (HRM) practitioners who needed to find ingenious solutions to sustain their company's business and to help their employees to cope with the challenges of this unprecedented situation. In this context, there are very few studies on the impact of COVID-19 on HRM, its challenges, and its potential opportunities for HRM in organizations, whereas managers and HRM practitioners need relevant information that will help them to go through this crisis effectively and efficiently, to be able to support their employees and to sustain their company's business. In fact, organizations are generally not sufficiently prepared to deal with crises when they occur (Wang, Hutchins, & Garavan, 2009). Whence the importance, for the scientific community, to support organizations by providing relevant information related to this new pandemic. Therefore, the principal goal of this research is to investigate the impact of COVID-19 on HRM, to identify the main challenges and opportunities, and to provide insights into future directions in HRM. From a scientific perspective, this paper aims at broadening the scope of management research, considering the scarcity of papers on this topic.

## Methodology

This paper is a general literature review, with an informative purpose, that aims to examine recent and relevant literature which investigated the impact of COVID-19 on HRM. There are very few studies that have investigated this impact. Thus, we have started to search for articles which examined generally the relationship between COVID-19 and HRM, then we searched for articles that examined the impact of this pandemic specifically on each HRM function and practice, e.g., staffing (recruitment) and compensation. We searched for articles in Google Scholar, Ebsco, and Semantic Scholar using a combination of terms related to coronavirus OR COVID-19; Human resource management; HRM; pandemic and HRM functions (e.g., compensation and staffing). The search for articles was performed manually. We searched for articles published between December 2019 and February 2021. We have excluded epidemiological articles. The articles analyzed in this paper are all listed in the section ‘References.’

## Literature review

### Human resource management facing COVID-19: implications and challenges

HRM ‘is about how people are employed, managed and developed in organizations’ (Armstrong & Taylor, 2020: 3). It has been grandly impacted by COVID-19, generating significant challenges for managers and HRM practitioners. This impact and these challenges are explored in this section, in relation to strategic HRM and working conditions, as well as HRM functions, specifically, staffing, performance management, training and development, compensation management, safety and health management, and employees' relations. Each HRM function is discussed individually, however, they are interrelated. This suggests that any change in one HRM function will affect the other function (Mondy & Martocchio, 2016).

### COVID-19 and strategic human resource management

Strategic HRM refers to the vertical connection between HRM functions and the organizational strategy as well as the horizontal consistency between HRM functions (Wright & McMahan, 1992). Its main purpose is to effectively utilize the human resources to serve the strategic needs of the organization (Chapman, Sisk, Schatten, & Miles, 2018; Navío-Marco, Solórzano-García, & Palencia-González, 2019; Schuler, 1992).

In order to ensure the achievement of the organizational goals in a time of crisis, strategic agility is required (Liu, Lee, & Lee, 2020). Organizations need to be able to prepare and allocate their resources; to coordinate the needed mechanism; and to properly use the organizational resources and knowledge (Liu, Lee, & Lee, 2020). In this context, the novelty and the complexity of the COVID-19 represent a significant challenge that might compromise the achievement of organizational goals. According to Baert, Lippens, Moens, Sterkens, and Weytjens (2020), standard economic models in organizations are mainly trained to use data from ‘normal times’ perspective. Thus, it is challenging to make predictions related to ‘abnormal times.’ This might suggest that making predictions related to the company's business, e.g., the preparation and the allocation of resources might be a complex exercise. In fact, COVID-19 has generated uncertainty. Some authors go so far as to predict the COVID-19 endemic (Regmi & Lwin, 2020) whereas many economists predict the outcomes of this pandemic will remain until 2021 (Akkermans, Richardson, & Kraimer, 2020). Currently, no one knows when this virus will end and if its consequences on the work patterns in organizations will be temporary or permanent (Bartik, Cullen, Glaeser, Luca, & Stanton, 2020), even after the recent development of different types of vaccines (Yu et al., 2021). Thus, performing strategic planning or implementing the initial one can be challenging for managers and HRM practitioners. In this case, most organizations were not able to provide their employees enough information about their management plan or their intended reactions toward the pandemic (Elsafty & Ragheb, 2020), whereas having clear workplace guidelines during hard times helps to reduce employees’ stress and to increase their motivation and confidence (Wong, Ho, Wong, Cheung, & Yeoh, 2020). The study by Elsafty and Ragheb (2020) showed that access to information and the update related to the pandemic is associated significantly with employees' retention. Nonetheless, it might be difficult to achieve it if organizations are not able to get this information, especially when they are in a reactive and survival mode, due to the novelty of this pandemic. Although challenging, enhancing organizational resilience is crucial to ensure the sustainability of the organization in the COVID-19 era (Ngoc Su, Luc Tra, Thi Huynh, Nguyen, & O'Mahony, 2021). In fact, despite the uncertainty generated by this pandemic, organizations need to develop ingenious practices that can help absorb and face disturbance that threatens their survival (Ngoc Su et al., 2021)

### Working conditions

Working conditions represent ‘the core of paid work and employment relationships’ (ILO, 2020). They ‘cover a broad range of topics and issues, from working time (hours of work, rest periods, and work schedules) to remuneration, as well as the physical conditions and mental demands that exist in the workplace’(ILO, 2020). The COVID-19 crisis has drastically altered working conditions in organizations. Indeed, to ensure their business continuity, most organizations have moved to remote working, requiring their employees to work from home (Aitken-Fox et al., 2020a, 2020b; Gourinchas, 2020; Koirala & Acharya, 2020). For example, Google announced that its employees will continue working remotely until at least Summer 2021 whereas Twitter's employees were given the opportunity to work remotely indefinitely (Leonardi, 2020). Notwithstanding, the category of employees working from home represents a small fraction of the overall workforce (Gourinchas, 2020), mainly because remote working is not suitable for manufacturing industries (Koirala & Acharya, 2020) and it cannot be applied to all job positions (Bartik et al., 2020). In this context, there were two possible scenarios for companies whose nature of their business does not allow them to adhere to these types of working conditions. Either to require their employees to be physically present while respecting the measures of physical distancing (i.e., allow an interval of 2 min between individuals) and wearing personal protective equipment or to lay them off (Blustein, Duffy, Ferreira, Cohen-Scali, Cinamon, & Allan, 2020). The study by Adams-Prassl, Boneva, Golin, and Rauh (2020) showed that employees whose job tasks cannot be performed from home are more likely to lose their jobs. In these circumstances, HRM practitioners are urged to identify the job positions that can be performed remotely, those which can be performed in the physical workplace, and those positions that need layoff due to the situation provoked by the pandemic. Therefore, these unexpected and drastic organizational changes represent significant challenges for managers and HRM practitioners. In addition, they might have significant implications on employees mental health (Hamouche, 2020) and person–environment fit perceived by employees (Carnevale & Hatak, 2020) as well as the employee experience related to the job design, the workspace and interactions with their peers and managers (Aitken-Fox et al., 2020a).

Physical presence in workplaces has been maintained with rigorous protection measures (e.g., physical distancing and wearing protection masks) with the implementation of work schedules for different groups of employees (Akbarpour et al., 2020). The principal challenge, in this case, is to ensure the respect of these protection measures and to plan work schedules that consider employees' context.

As for remote working, it seems that managers and HRM practitioners have faced major challenges. First, to ensure that employees working from home have the necessary tools to perform their job (Aitken-Fox et al., 2020b; Hamouche, 2020). Actually, remote working requires the availability of technological tools which will facilitate communication between employees and managers, such as Zoom, Microsoft remote desktop, team viewer, and Microsoft team (Prasad & Vaidya, 2020), that cannot be afforded by all organizations, considering that the financial capacity varies from one organization to another. Second, to ensure for the employees working from home effective communication, supervision, support, performance management, and a realignment of their compensation (Aitken-Fox et al., 2020b). Moreover, HRM practitioners need to support managers who are leading remote teams for the first time (Caligiuri, De Cieri, Minbaeva, Verbeke, & Zimmermann, 2020). Finally, HRM practitioners need to take into consideration the fact that remote working might lead to employees' isolation due to the absence of interaction between employees, lack of peer advice, and lack of one-to-one communication which can be sources of stress that might undermine employees' mental health (Prasad & Vaidya, 2020). It can also be psychologically demanding for these employees considering the possibilities of family distractions and the multiple roles that they have to assume while working from home (Prasad & Vaidya, 2020). In addition, the increased use of information and communication technology (ICT) can lead to the perception of an everlasting urgency, generating possible expectations about the constant availability of employees (Molino et al., 2020). Working from home can lead, as well, to an increase in the volume of information treated by employees, considering that they regularly use their emails (Leonardi, 2020). Some authors refer to a technostress related to the use of ICT, which has increased among employees working remotely (Molino et al., 2020). This can undermine the psychological health of employees, especially those who isolate themselves by choosing only emails as a means of communication.

Many HRM practitioners have implemented some activities to support their employees, such as creating virtual socialization activities, e.g., virtual lunch or coffee breaks (Carnevale & Hatak, 2020; Maurer, 2020). Undoubtedly, these practices help to support employees in this tough crisis while they are far from each other, and from their workplace (Hamouche, 2020). However, they also represent a great challenge for organizations, considering that besides being applied in a context of unexpected changes, these practices are new for employees and managers, who have not been previously trained or psychologically prepared for such changes, which may lead to an increase in their perceptions of person–environment misfit and dissatisfaction if they prefer the face-to-face interactions that they used to have prior to this pandemic outbreak (Carnevale & Hatak, 2020). Moreover, virtual interactions might affect the socialization process recognized for its importance to help employees acquire the tacit knowledge related to the organizational culture, and contributing to its development (Asatiani, Hämäläinen, Penttinen, & Rossi, 2021)

### Staffing

Staffing refers to ‘ the process of attracting, selecting, and retaining competent individuals to achieve organizational goals’ (Ployhart, 2006: 868, 868). It had been greatly impacted by COVID-19, which has reshaped its dynamic in organizations (Campello, Kankanhalli, & Muthukrishnan, 2020).

COVID-19 had mostly asymmetric impacts on industries (Aitken-Fox et al., 2020b; Giupponi & Landais, 2020). Some industries were experiencing a sharp decline in their business (Giupponi & Landais, 2020) leading some of them to temporarily close their shops (Bartik et al., 2020), whereas other industries have seen their business flourishing during this pandemic (Giupponi & Landais, 2020). Therefore, the repercussions of COVID-19 on staffing differ from one organization to another.

In this context, organizations that were facing financial difficulties due to this pandemic have adopted downskilling by cutting back on recruitment of high-skill jobs more than low-skill jobs, to reduce their costs and try to sustain their business (Campello, Kankanhalli, & Muthukrishnan, 2020); they have frozen or cut back all their recruitment; or they have laid off their employees (Campello, Kankanhalli, & Muthukrishnan, 2020; Giupponi & Landais, 2020). Indeed, millions of people found themselves unemployed due to the COVID-19 outbreak (Blustein et al., 2020; Elsafty & Ragheb, 2020). Cheng et al. (2020) pointed out that the employment activities have increased after the companies' reopening in some US states mainly due to the return to work of employees, after lockdown, to their physical workplace. Nevertheless, the reemployment probabilities diminish significatively for employees who stayed longer away from their workplace.

Laying off employees is not an easy decision for organizations, but it might be inevitable in times of crisis such as COVID-19. The main challenge of HRM practitioners, in this case, is to support managers and employees during this process and to offer proper information. However, it might not be easy in the context of uncertainty. Actually, all over the world, no one knows when this pandemic will end and if its consequences on organizations will be temporary or permanent (Bartik et al., 2020).

On the contrary, organizations that have expanded their business during the pandemic have faced other types of staffing challenges. Many of them have opted out for more flexible employment relationships and subcontracted work, such as temporary agency work, freelancers, and the gig economy (Spurk & Straub, 2020), due to uncertainty generated by COVID-19.

Indeed, these organizations have increased their recruitment (Akkermans, Richardson, & Kraimer, 2020; Giupponi & Landais, 2020), nonetheless, they found themselves facing the pressure of workforce shortage (Giupponi & Landais, 2020). In fact, how to recruit employees when people are afraid of contagion? How to select employees when it is not allowed to meet them face to face, due to the physical distancing measures? In these circumstances, these organizations had no other choice than to orient their practices toward virtual recruitment and selection methods (Carnevale & Hatak, 2020; Maurer, 2020), which might represent another significant challenge for HRM practitioners as well as job applicants. Not all individuals are comfortable using ICT tools. Also, HRM practitioners were not prepared for this type of unexpected change. Moreover, virtual selection methods might affect the ability of potential employees and employers to assess person–environment fit, which can have a negative impact on employees' productivity and retention (Carnevale & Hatak, 2020). Besides, the temporary character of flexible employment relationship posed the challenge of employees' retention.

According to some authors, employee retention might represent another major challenge for organizations in the current context of this pandemic (Elsafty & Ragheb, 2020; Ngoc Su et al., 2021). Elsafty and Ragheb (2020) pointed out that during these hard times characterized by drastic and sudden changes, employment relationships might be damaged, leading to the possible dramatic decrease of employees' morale and an increase in turnover. Furthermore, according to Ngoc Su et al. (2021) retaining and attracting qualified individuals represent a challenge for companies in the COVID-19 era, mainly because these individuals are often looking for job opportunities in sectors that were not negatively affected by this pandemic. In the same vein, Przytuła, Strzelec, and Krysińska-Kościańska (2020) highlighted the importance to increase the engagement and the sense of belonging among employees, mainly the remote workforce during this period and beyond, to ensure organizational success, and prevent recruitment costs (Lund et al., 2021).

### Performance management

Performance management is ‘a continuous process of identifying, measuring, and developing the performance of individuals and workgroups and aligning performance with the strategic goals of the organization’ (Aguinis, 2019: 8). It is crucial to ensure that employees' performance is aligned with the company's strategic goals (Ismail & Gali, 2017).

To sustain the company amid a crisis like COVID-19, employees are still required to maintain their good performance (Sembiring, Fatihudin, Mochklas, & Holisin, 2020). However, it seems that the COVID-19 outbreak has also altered performance management in organizations. According to some authors, most organizations were overwhelmed by the challenges resulting from COVID-19, such as measuring employees' performance and the disruption in performance-based pay, that they have reduced or even abandoned performance management, due to the complexity and the novelty of this pandemic (Aguinis & Burgi-Tian, 2020). In fact, measuring employees' performance during this crisis can be challenging, considering the modification of the working conditions. Furthermore, there are many factors related to the COVID-19 outbreak that may influence employees' performance. In this context, the study by Prasad and Vaidya (2020) reported that workplace isolation, lack of communication, family distractions, role overload, and occupational stress factors (role ambiguity, role conflict, career, and job-control), which have emerged due to COVID-19, mainly among employees working from home are significant predictors of employees' performance. Furthermore, employees' performance during remote working is also dependent on managers' understanding of how and what is required to manage a remote team (Aitken-Fox et al., 2020b). Some authors argued that managers might not accept remote working because they might consider that it affects employees' performance negatively, which can lead to the adoption of micromanagement that can be perceived by employees as a lack of trust toward them (Aitken-Fox et al., 2020b), which can create tension between them and their supervisor.

According to Aguinis and Burgi-Tian (2020), it is crucial for organizations during this health crisis to maintain and strengthen their performance management process. They should communicate relevant information related to the company's strategic direction to their employees, to collect useful business data, and to provide feedback to them, which will help these organizations to retain their talents and to avoid legal suits. Ngoc Su et al. (2021) added that the frequent appraising of employees' performance fosters their learning and sharing that can help organizations to win back their business. Considering the interrelation between HRM functions, the study by Sembiring et al. (2020) showed that compensation might have a significant impact on employees' performance in the COVID-19 era. Hence, the authors suggested that organizations should be more concerned about employees' total compensation (financial and non-financial), and its fairness to sustain and improve their performance during crises (Sembiring et al., 2020). The main challenge, in this context, might be related to the financial capacity of the organization during this ongoing pandemic.

### Training and career development

Training plays an important role in a period of crisis, such as pandemics (Devyania, Jewanc, Bansal, & Denge, 2020; Hamouche, 2020). It helps to develop the needed skills for employees (Akkermans, Richardson, & Kraimer, 2020); to increase the COVID-19 awareness, to reduce the risk of the virus spread, and to prevent mental health issues (Quaedackers et al., 2020). It also helps to support employees in the process of transition toward remote working. In fact, not all employees have the proper digital skills to cope with these changes generated by the use of ICT, whence the necessity to train them on the utilization of ICT, which will help to facilitate their work and communication with their manager and peers while they are away from their workplace (Greer & Payne, 2014). According to Przytuła, Strzelec, and Krysińska-Kościańska (2020), organizations face the challenge of reskilling and upskilling their workforce to be able to deal with the requirement of new context of ‘ distance economy.’ In this case, the main challenge for HRM practitioners might be related to the development of a training program adapted to the new reality of the organization and the employees and to choose the proper training methods, considering physical distancing measures coupled with the necessity to have employees quickly operational to sustain the company business. This suggests that managers and HRM practitioners need to go beyond the traditional training methods. Devyania et al. (2020) recommended, in this case, to change employees' training programs in a way that ensures a long-term transition toward the new working practices.

The success of remote working is also dependent on managers' understanding of the virtual supervision of employees (Aitken-Fox et al., 2020b). In this context, the HRM practitioners should play a strategic role by supporting and training these managers on how to manage a virtual team, to help them to overcome these difficulties and to cope with remote working challenges in order to be able to support their team members (Hamouche, 2020).

Besides training, COVID-19 has posed significant challenges related to career development in organizations. According to some authors, COVID-19 has led to a grand career shock (Akkermans, Richardson, & Kraimer, 2020; Baert et al., 2020). The study by Baert et al. (2020) based on the analysis of the impact of COVID-19 on career outcomes and aspiration among a panel of 3,821 employees, showed that due to the COVID-19 crisis, employees were afraid of losing their job in the near future. In addition, some of them expected to miss out on a promotion that they should have received if this crisis has not happened.

### Compensation management

Compensation management refers to the intrinsic and extrinsic rewards that employees receive for performing their job. It encompasses monetary (base pay/bonuses) and non-monetary rewards (employee benefits) (Martocchio, 2017). Compensation can influence employees' motivation, performance (Safuan & Kurnia, 2021; Sembiring et al., 2020), and retention (Elsafty & Ragheb, 2020). The study by Elsafty and Ragheb (2020) showed that financial benefits such as bonuses during COVID-19 are associated significantly with employees' retention.

As a reaction to the COVID-19 outbreak, some countries have implemented governmental policies to provide financial support for employees and organizations during this health crisis and to encourage them to comply with the stay-at-home orders. For instance, in the USA, the federal government has enacted the temporary paid sick leave, allowing private and public sectors employees 2 weeks of paid sick leave for isolation, treatment related to COVID-19, taking care of a member of their family infected by COVID-19, and childcare caused by the school or daycare closure (Andersen, Maclean, Pesko, & Simon, 2020). Short-time compensation, also known as part-time jobs, has also been adopted to sustain the economy while protecting business and employees' jobs. It consists of offering employees a temporary reduction in the number of their working hours which will help organizations that are experiencing a decrease in the level of demand, to retain their employees and to avoid layoffs (Giupponi & Landais, 2020). These measures alter compensation strategies and policies within organizations. Furthermore, they might create a complex and challenging environment for managers and HRM practitioners. According to some authors, paid sick leave might lead to an increase in employees' absence in the workplace (Maclean, Pichler, & Ziebarth, 2020). But at the same time, it helps to prevent employees' presenteeism when they are sick (Schneider, 2020). Additionally, this type of government's policies, such as paid sick leave, help to increase their implementation in industries where employees have never got such benefits (Maclean, Pichler, & Ziebarth, 2020), which suggest that managers and HRM practitioners need to think about the way to sustain them to avoid losing employees' motivation after the pandemic. In this context, Przytuła, Strzelec, and Krysińska-Kościańska (2020) referred to the importance of intrinsic motivation to retain employees, e.g., increasing employee autonomy.

Furthermore, compensation management can be particularly challenging in workplaces where the risk of contamination is very high, for example in hospitals. In this context, the level of compensation offered to employees may be questioned, to know if it is high enough considering the level of risk that these employees encounter daily (Hecker, 2020). According to Hecker (2020), individuals use to select jobs based on their risk tolerance in return for more compensation for higher risks. Generally, the employer's intervention is oriented toward the necessary control of hazards to be able to recruit individuals for job positions with higher risks. Hence, in case of a high level of risk associated with the job position without sufficient compensation, many employees might decide to leave the organization (Hecker, 2020).

### Safety and health management

Employers are responsible for the protection of their employees while they are working. They must ensure that the workplace is free from any hazard that may psychologically or physically harm them or cause their death. COVID-19 has generated a new workplace hazard (Hecker, 2020) that represents a significant source of stress for employees (Shaw et al., 2020) and a significant challenge for managers and HRM practitioners (Hamouche, 2020). The impact on employees' health varies based on the working environment and the employee's occupational role (Brooks, Dunn, Amlôt, Rubin, & Greenberg, 2018). Two main challenges can be identified in this context: how to control the spread of the virus and to protect employees from contagion and how to develop the employees' awareness about the importance to respect the prevention measures implemented in the workplace. The WHO has provided guidelines for organizations to ensure the protection of their employees (WHO, 2020a), nonetheless, controlling employees' behavior might be challenging, considering that some people may ignore self-isolation instructions (Gourinchas, 2020).

The recent development of vaccines against COVID-19 has brought the light of hope all over the world, but it has also generated two additional new challenges for organizations, specifically the management of the vaccination campaign in the workplace as well as their capacity to sponsor it and cover its costs (Rothstein, Parmet, & Reiss, 2021), considering the financial difficulties that they have witnessed due to this pandemic.

COVID-19 is not only a physical health risk, but it also represents a significant risk for individuals' mental health (Brooks et al., 2020; Chen, Ning, Yu, Huang, Li, & Luo, 2020; Hamouche, 2020; Qiu, Shen, Zhao, Wang, Xie, & Xu, 2020). It might be psychologically demanding for employees who work from home, who can feel isolated and torn between their work and their private life (Prasad & Vaidya, 2020). Moreover, employees who are required to be physically present in the workplace might return to work with the fear of contracting the virus or transmitting it to their family (Tan et al., 2020), which might increase their level of stress as well as the risk of mental health issues (Hamouche, 2020), especially for employees who were facing high psychological demands at work, prior to the pandemic (Quaedackers et al., 2020), or those who have a high-risk job position, e.g., healthcare workers (Hamouche, 2020). The main challenge for managers and HRM practitioners, in this context, is to identify the risk factors and to implement the proper prevention measures in the workplace, including for employees working from home (Hamouche, 2020).

### Employment relationship

Employment relationship refers to ‘the connection between employees and employers through which individuals sell their labor’ (Budd & Bhave, 2010). From a labor law perspective, COVID-19 has created important challenges for employees and employers (Biasi, 2020; Sagan & Schüller, 2020). Due to the lockdown and mandatory closure of business both were not able to accomplish their contractual obligations (Biasi, 2020). In fact, the challenges resulting from COVID-19 have transformed the traditional relationship between the employee and his employer (Leighton & McKeown, 2020; Spurk & Straub, 2020). Work from home has been implemented in different countries and companies (Spurk & Straub, 2020). Hence, the traditional boundaries of the world of work have disappeared (Leighton & McKeown, 2020). In this context, COVID-19 has positioned the government as a planner more than a regulator (Sachs, 2020), which is challenging for organizations that need to adapt government plans and regulations to their organizational context, while taking into consideration the needs of their employees (Sachs, 2020).

Considering the novelty of this pandemic, most countries do not only rely on existing regulations. They have amended, over a short and a prompt period, several labor laws (Sagan & Schüller, 2020), to support employers and to protect employees (Alhambra, 2020; Mangan, 2020; Sachs, 2020; Sagan & Schüller, 2020). The main challenge was how to protect employees while ensuring the continuity of the economy (Sachs, 2020). Some countries have adopted laws to structure and temporarily prohibit collective layoff in organizations (Biasi, 2020). For example, in Italy, a decree law has been issued to prohibit organizations from initiating a collective layoff procedure for a period of 60 days (Biasi, 2020). Furthermore, various legal measures and laws have been adopted to support employees during the lockdown and closure of schools, e.g., employees were given paid leave to take care of their children. The main challenge is the fact that it is still unclear when countries can declare the ‘end’ of this pandemic (Spurk & Straub, 2020). This represents a critical challenge for determining the proper period of protection needed by employees, which might undermine the relationship between organizations, employees, and their representatives (Biasi, 2020). COVID-19 is an exceptional crisis that has generated extraordinary measures. In some countries, e.g., in France, remote working is voluntary and cannot be imposed by employers (Sachs, 2020), however, as in many other countries the current situation has led employers to impose this mode of working on employees whose job position can be performed from home (Sachs, 2020). The main challenge, in this case, is the fact that disputes might arise between employers and the employees who had not been offered the possibility to work from home or have contracted COVID-19 at the workplace (Sachs, 2020). In fact, in case of a lack of contractual agreement, it is possible, according to Sagan and Schüller (2020), to question the consistency of the employers with the labor laws.

Currently, with the recent development of COVID-19 vaccines, the main challenge for organizations from an employment relationship perspective is the management of the vaccination campaign in terms of costs and application, while ensuring compliance with the country regulations (Rothstein, Parmet, & Reiss, 2021). This development also raises the question about the ability of the employer to impose it on their employees. According to Rothstein, Parmet, and Reiss (2021), the adoption of a rigid, coercive approach could intensify the reluctance of the individuals who are not sure yet about the vaccine. These authors suggested that organizations should educate their employees about the benefits of vaccination and should facilitate it, for example by offering time off for employees for vaccination purposes, rather than imposing it (Rothstein, Parmet, & Reiss, 2021).

## Opportunities, future organizational directions, and insights into HRM interventions

COVID-19 has posed grand challenges for managers and HRM practitioners, but it has also opened the door to opportunities worth knowing and understanding, that can help organizations to direct their future actions. Indeed, according to Demirkaya and Aydın (2006), a crisis might create unexpected opportunities for organizations. In this section, we will discuss these opportunities while linking them to the potential future directions in HRM.

COVID-19 has challenged organizations' creativity and innovation and has urged discussions about the future of work (Hite & McDonald, 2020). It has accelerated the disruption of HRM as well as the implementation of scenarios expected for the future (Hite & McDonald, 2020). Moreover, it has pushed organizations to rethink their HRM strategies and to go beyond the traditional models of managing human resources, by positioning new information technology as an essential partner to survive and to ensure the sustainability of their business. In this context, new legislation has been adopted in different countries to support organizations in this sudden and unexpected transformation. For example, Germany has adopted new legislation to introduce the possibility of video conferencing in two areas (Sagan & Schüller, 2020), to support the implementation of remote working in organizations.

Therefore, the normality that seems to be emerging for the moment in workplaces is the implementation of remote working. However, it is earlier, according to some authors, to confirm that all organizations which have adopted remote working will continue to adopt it in the future, beyond COVID-19 (Aitken-Fox et al., 2020b). They are probably reviewing the effectiveness of this work organization before taking their decision, considering that they have implemented it for the first time. Therefore, they do not know yet how it can affect employees' performance and productivity (Aitken-Fox et al., 2020b). However, it seems according to a recent report published by McKinsey Global Institute which assessed the lasting impact of COVID-19 on labor demands, occupations, and workforce skills in eight countries (China, France, Germany, India, Japan, Spain, the United Kingdom, and the United States) that remote work, as well as virtual meetings, will continue but less intensely than at the peak of COVID-19 (Lund et al., 2021).

Despite its challenges, remote working offers employees the opportunity to have flexible working hours, save commuting time, foster job control, and experience the use of new ICT (Prasad & Vaidya, 2020). In addition, it offers companies the opportunity to optimize the use and save the costs of their resources, e.g., office space. Actually, business sectors in some countries, for example in Korea, see growth opportunities in non-contact industries which encompasses telecommunication, remote support solutions, and online education (Liu, Lee, & Lee, 2020).

Moreover, COVID-19 offers opportunities for organizations to develop the autonomy of their employees, upgrade their digital competencies, and broaden the perspective of their competencies' development. Besides, this pandemic has positioned new technology as a strategic partner for organizations. It has helped to sustain businesses and to shorten the distance between employees and their employers while ensuring their safety. It has fostered the creativity of managers and HRM practitioners and it has facilitated the transition from traditional face-to-face socialization methods to virtual ones, e.g., virtual meetings, lunches, and coffee breaks (Carnevale & Hatak, 2020). It has also helped to sustain staffing in organizations while respecting physical distancing measures.

The new technology has also supported the management of safety and health in workplaces. It has helped to implement the decision to keep the employees at home and to protect them from the risk of infection, while they keep on working for the organization. It has also supported healthcare professionals, e.g., psychologists who have continued to help the population through telehealth systems while respecting physical distancing measures. In China, for instance, mental health services have been provided, during the pandemic, using various channels such as hotlines, online consultations, online courses (Gao et al., 2020), and telemental health services (Zhou et al., 2020). According to Lund et al. (2021), COVID-19 may accelerate the adoption of automation and artificial intelligence (AI) in sectors with high levels of human interaction, such as medical care and personal care (e.g., gyms and hair salons). Hence, it is crucial for organizations to get to grips with ICT and to make it accessible to all its members, in order to be able to sustain their business during extraordinary crises. Some authors insisted on the importance of using AI for HRM during a period of a health crisis and recommended using it as an effective tool to prevent disruptions in operations and management practices while ensuring physical distancing and the protection of employees (Devyania et al., 2020). In the same vein, Liu, Lee, and Lee (2020) recommended the development of predictive models, which takes into account the risk factors and the uncertainties in the proactive scheduling and planning of supply, which might help decision makers to create various dynamic scenarios that can be automatized with the use of AI. The use of new technology also supports data analytics that can help HRM practitioners to optimize and improve HRM functions and practices in organizations, such as workforce planning, recruitment, and talent management (AM, Affandi, Udobong, & Sarwani, 2020), during this pandemic and beyond.

Identifying the opportunities generated by COVID-19 can help HRM practitioners to develop the proper HRM interventions and future actions. Nevertheless, it is important to take into account the fact that organizations all over the world are still witnessing the pervasive effect of this pandemic that does not seem to end quickly. Undoubtedly, the enhancement of organizational resilience is required. In this context, organizations need to be able to develop innovative responses to effectively absorb and face disturbance that threatens their survival (Ngoc Su et al., 2021). HRM practitioners should work in collaboration with managers and employees to transform the challenges brought on by COVID-19 into opportunities, to rethink their HRM functions and practices, e.g., compensation and performance management, and to adapt them to the employees' new working conditions generated by the COVID-19 crisis. According to Przytuła, Strzelec, and Krysińska-Kościańska (2020), organizations need to lay new foundations, by redefining the new trends in HRM practices. In fact, after almost more than 1 year of new functioning based mostly on remote work, organizations need to re-evaluate their context, compare the new trends in HRM generated by this unexpected crisis, and assess their applicability.

Considering the unpredictability of the current situation and the high level of doubt surrounding its end, organizations should opt out to move toward a hybrid workplace model (AM et al., 2020; Kaufman, Lovich, Bailey, Messenböck, Schuler, & Shroff, 2020; Przytuła, Strzelec, & Krysińska-Kościańska, 2020), flexible enough to allow a quick and efficient adaptation of the organization to the requirements of this new situation and beyond. HRM practitioners need to adapt job positions and focus on job redesign within the organization. Lund et al. (2021) suggested, in this case, emphasizing necessary tasks and activities related to a job rather than the whole job to increase the organizations' operational flexibility as well as agility. Employees should receive the necessary organizational support to acquire the skills needed during this pandemic and beyond, coupled with the development of career pathways offering possibilities of upward mobility (Ngoc Su et al., 2021) and enhancing their employability. Such interventions should have a positive impact on employees' motivation and retention as well as the reduction of the costs related to recruitment (Lund et al., 2021). Some authors go so far as to suggest that organizations should reinvent themselves by the integration of entrepreneurship competencies among their employees, to help them to learn how to adjust themselves to the uncertainty that can be generated by an unexpected crisis and to thrive in a dynamic environment (Carnevale & Hatak, 2020; Liu, Lee, & Lee, 2020). The field of entrepreneurship might help employees to explore, to evaluate, and to exploit opportunities that occur in a dynamic and unstable environment, considering that this field is based on exploration, evaluation, discovery, and the capacity to transform challenges brought on by an ambiguous context into opportunities (Carnevale & Hatak, 2020).

Besides, the pivotal role of the new information technology during the pandemic should urge managers and HRM practitioners to explore effective ways to integrate it into HRM and adapt it to the context of their organization. Moreover, they need to identify the specific training needs, as not all employees, including managers, have the proper technological competencies. The involvement of employees is required to ensure the success of this organizational change. Additionally, sustaining communication with them should help to reduce their stress and increase their trust in the organization (Hamouche, 2020).

Furthermore, employees should be given the possibility to work remotely with the flexibility to choose when and where to work (Kaufman et al., 2020; Przytuła, Strzelec, & Krysińska-Kościańska, 2020), without limiting the workspace to their home. However, organizations should provide the possibility to schedule a flexible presence in the office to keep the employees connected to their workplace, by ensuring the presence of efficient health and safety measures and facilitating access to vaccination. Considering the blur surrounding private and professional life boundaries, managers should communicate and discuss with their employees the expectations of the organizations in terms of performance. In this regard, managers, supported by HRM practitioners, should review and realign the performance management system in order to adapt the performance objectives to the new reality of organizations and employees (AM et al., 2020). They should provide continuous feedback that will enhance learning and sharing among employees and foster organizational flexibility, agility as well as employees' motivation and retention (Ngoc Su et al., 2021). They should also develop wellbeing programs that aim at protecting employees' mental health, and providing solutions adapted to the needs of every employee, in terms of resources and social support (Hamouche, 2020). Managers should discuss with their employees the different scenarios of work schedules adapted to the requirements of the current situation. Also, with the support of HRM practitioners, managers should increase employees' awareness about the necessity to disconnect from work when it is required to prevent mental health issues.

In this context, rebuilding the organizational culture is needed to facilitate the adoption of flexible work arrangements and the transition toward a hybrid working model (AM et al., 2020; Ngoc Su et al., 2021). Moreover, developing and maintaining a cohesive culture that supports employees' connections and interactions is required (Lund et al., 2021), to encourage social support and collaboration among employees, particularly those working remotely. In fact, virtual interactions might affect the socialization process, the acquisition of tacit knowledge related to the organizational culture (Asatiani et al., 2021). This led some authors to recommend the development and implementation of a digital organizational culture handbook, which should be made available to employees working remotely through the organization's intranet, to provide toolkits that support and vehicles symbolic aspects of the organizational culture, such as values (Asatiani et al., 2021).

## Contribution and practical implications for organizations

In the business world, crises are inevitable. However, no one can predict a crisis with the magnitude of COVID-19, which has accelerated the disruption of traditional methods of HRM and has created significant challenges for managers and HRM practitioners, who were not fully equipped in terms of information, resources, and competencies to cope with the complexity and the novelty of this pandemic.

Besides these challenges, COVID-19 has opened the door to opportunities that organizations should know to be able to properly direct their future actions in HRM. This paper is a general literature review that provides relevant and useful information which can help managers and HRM practitioners to understand the main challenges and opportunities related to COVID-19. The insights provided in this paper into the future directions in HRM should help them to develop an intervention plan adapted to the needs of their organizations and employees.

## Conclusion and future research

The contribution of this paper should, however, be considered in light of some limitations. First, our research is a general literature review with an informative purpose, which might suggest that there is a possibility of a subjective selection of literature. Notwithstanding, the databases that we have used (Google Scholar, Ebsco, and Semantic Scholar) provide the most cited articles. Besides, the purpose and the informative character of this paper do not require a systematic review of the literature. Second, during writing this paper, COVID-19 is still present. Therefore, it is not possible to identify accurately the long-term challenges and opportunities. Future research should be directed toward longitudinal analysis to identify these challenges and opportunities.

## References

[ref1] Adams-Prassl, A., Boneva, T., Golin, M., & Rauh, C. (2020). Inequality in the impact of the coronavirus shock: Evidence from real time surveys. Journal of Public Economics, 189, 104245.

[ref2] Aguinis, H. (2019). Performance management for dummies. New Jersey: John Wiley & Sons.

[ref3] Aguinis, H., & Burgi-Tian, J. (2020). Measuring performance during crises and beyond: The performance promoter score. Bus Horiz, 64(1), 149–160. 32981944 10.1016/j.bushor.2020.09.001 PMC7505577

[ref4] Aitken-Fox, E., Coffey, J., Dayaram, K., Fitzgerald, S., Gupta, C., McKenna, S., & Wei Tian, A. (2020a). COVID-19 and the changing employee experience. LSE Business Review. Retrieved from https://blogs.lse.ac.uk/businessreview/2020/06/24/covid-19-and-the-changing-employee-experience/

[ref5] Aitken-Fox, E., Coffey, J., Dayaram, K., Fitzgerald, S., Gupta, C., McKenna, S., & Wei Tian, A. (2020b). The impact of Covid-19 on human resource management: avoiding generalisations. LSE Business Review. Retrieved from https://blogs.lse.ac.uk/businessreview/2020/05/22/the-impact-of-covid-19-on-human-resource-management-avoiding-generalisations/

[ref6] Akbarpour, M., Cook, C., Marzuoli, A., Mongey, S., Nagaraj, A., Saccarola, M., … Yang, H. (2020). Socioeconomic Network Heterogeneity and Pandemic Policy Response. University of Chicago, Becker Friedman Institute for Economics Working Paper (2020-75).

[ref7] Akkermans, J., Richardson, J., & Kraimer, M. (2020). The Covid-19 crisis as a career shock: Implications for careers and vocational behavior. Journal of Vocational Behavior, 119, 1–6. doi: 10.1016/j.jvb.2020.103434 PMC7205633 32390655

[ref8] Alhambra, M. A. G.-M. (2020). Covid-19 and labour law in Spain. European Labour Law Journal, 11(3), 319–323. doi: 10.1177/2031952520934576

[ref9] AM, E. N., Affandi, A., Udobong, A., & Sarwani, S. (2020). Implementation of human resource management in the adaptation period for new habits. International Journal of Educational Administration, Management, and Leadership, 1, 19–26.

[ref10] Andersen, M., Maclean, J. C., Pesko, M. F., & Simon, K. I. (2020). Effect of a federal paid sick leave mandate on working and staying at home: Evidence from cellular device data (0898-2937). Retrieved from https://www.nber.org/papers/w27138

[ref11] Armstrong, M., & Taylor, S. (2020). Armstrong's handbook of human resource management practice. USA: Kogan Page Publishers.

[ref12] Asatiani, A., Hämäläinen, J., Penttinen, E., & Rossi, M. (2021). Constructing continuity across the organisational culture boundary in a highly virtual work environment. Information Systems Journal, 31(1), 62–93.

[ref13] Baert, S., Lippens, L., Moens, E., Sterkens, P., & Weytjens, J. (2020). How do we think the COVID-19 crisis will affect our careers (if any remain)?, GLO Discussion Paper, No. 520, Global Labor Organization (GLO), Essen.

[ref14] Bartik, A. W., Cullen, Z. B., Glaeser, E. L., Luca, M., & Stanton, C. T. (2020). What jobs are being done at home during the COVID-19 crisis? Evidence from firm-level surveys (No. w27422). National Bureau of Economic Research.

[ref15] Biasi, M. (2020). Covid-19 and labour law in Italy. European Labour Law Journal, 2031952520934569.

[ref16] Blustein, D. L., Duffy, R., Ferreira, J. A., Cohen-Scali, V., Cinamon, R. G., & Allan, B. A. (2020). Unemployment in the time of COVID-19: A research agenda. doi: 10.1016/j.jvb.2020.103436 PMC7206417 32390656

[ref17] Brodeur, A., Gray, D. M., Islam, A., & Bhuiyan, S. (2020). A literature review of the economics of COVID-19, GLO Discussion Paper, No. 601, Global Labor Organization (GLO), Essen.

[ref18] Brooks, S. K., Dunn, R., Amlôt, R., Rubin, G. J., & Greenberg, N. (2018). A systematic, thematic review of social and occupational factors associated with psychological outcomes in healthcare employees during an infectious disease outbreak. Journal of Occupational and Environmental Medicine, 60(3), 248–257. doi: 10.1097/jom.0000000000001235 29252922

[ref19] Brooks, S. K., Webster, R. K., Smith, L. E., Woodland, L., Wessely, S., Greenberg, N., & Rubin, G. J. (2020). The psychological impact of quarantine and how to reduce it: Rapid review of the evidence. The Lancet, 395(10227), 912–920. doi: 10.1016/S0140-6736(20)30460-8. PMC7158942 32112714

[ref20] Budd, J. W., & Bhave, D. (2010). The employment relationship. In Wilkinson (Ed.),Sage handbook of human resource management (pp. 51–70). London: Sage.

[ref21] Caligiuri, P., De Cieri, H., Minbaeva, D., Verbeke, A., & Zimmermann, A. (2020). International HRM insights for navigating the COVID-19 pandemic: Implications for future research and practice. Journal of International Business Studies, 51, 697–713. 10.1057/s41267-020-00335-9 PMC7266413 32836500

[ref22] Campello, M., Kankanhalli, G., & Muthukrishnan, P. (2020). Corporate hiring under Covid-19: Labor market concentration, downskilling, and income inequality (No. w27208). National Bureau of Economic Research.

[ref23] Carnevale, J. B., & Hatak, I. (2020). Employee adjustment and well-being in the era of COVID-19: Implications for human resource management. Journal of Business Research, 116, 183–187. 32501303 10.1016/j.jbusres.2020.05.037 PMC7241356

[ref24] Chapman, E. F., Sisk, F. A., Schatten, J., & Miles, E. W. (2018). Human resource development and human resource management levers for sustained competitive advantage: Combining isomorphism and differentiation. Journal of Management & Organization, 24(4), 533–550.

[ref25] Chen, C., Ning, X., Yu, F., Huang, Q., Li, X., & Luo, Y. (2020). The mental health of neurological doctors and nurses in Hunan Province, China, during the COVID-19 outbreak. Research Square, 1, 1-11. doi: 10.21203/rs.3.rs-22061/v1 10.1186/s12888-020-02838-z PMC7474327 32891124

[ref26] Cheng, W., Carlin, P., Carroll, J., Gupta, S., Rojas, F. L., Montenovo, L., … Simon, K. I. (2020). Back to business and (Re) employing workers? Labor market activity during state COVID-19 reopenings.(No. W27419). National Bureau of Economic Research.

[ref27] Demirkaya, H., & Aydın, A. (2006). The strategic management and reorganization of human resource management in crisis process. Paper presented at the International Strategic Management Conference, Dedeman Oteli, İstanbul.

[ref28] Devyania, R. D., Jewanc, S. Y., Bansal, U., & Denge, X. (2020). Strategic impact of artificial intelligence on the human resource management of the Chinese healthcare industry induced due to COVID-19. IETI Transactions on Economics and Management, 1(1), 19–33. doi: 10.6897/IETITEM.202007_1(1).0002

[ref29] Elsafty, A. S., & Ragheb, M. (2020). The role of human resource management towards employees retention during Covid-19 pandemic in medical supplies sector – Egypt. Business and Management Studies, 6(2), 5059–5059.

[ref30] Gao, J., Zheng, P., Jia, Y., Chen, H., Mao, Y., Chen, S., … Dai, J. (2020). Mental health problems and social media exposure during COVID-19 outbreak. PLoS One, 15(4), e0231924. 32298385 10.1371/journal.pone.0231924 PMC7162477

[ref31] Giupponi, G., & Landais, C. (2020). Building effective short-time work schemes for the COVID-19 crisis. VoxEU,CEPR Policy Portal. Retrieved from https://voxeu.org/article/building-effective-short-time-work-schemes-covid-19-crisis

[ref32] Gourinchas, P. -O. (2020). Flattening the pandemic and recession curves. Mitigating the COVID Economic Crisis: Act Fast and Do Whatever, 31, 1–227.

[ref33] Greer, T. W., & Payne, S. C. (2014). Overcoming telework challenges: Outcomes of successful telework strategies. The Psychologist-Manager Journal, 17(2), 87. doi: 10.1037/mgr0000014

[ref34] Hamouche, S. (2020). COVID-19 and employees’ mental health: Stressors, moderators and agenda for organizational actions. Emerald Open Research, 2(15), 15. doi: 10.35241/emeraldopenres.13550.1

[ref35] Hecker, S. (2020). Hazard pay for COVID-19? Yes, but it's not a substitute for a living wage and enforceable worker protections. New Solutions: A Journal of Environmental and Occupational Health Policy, 30(2), 95–101. doi: 10.1177/1048291120933814 32567480

[ref36] Hite, L. M., & McDonald, K. S. (2020). Careers after COVID-19: Challenges and changes. Human Resource Development International, 23(4), 427–437.

[ref37] ILO. (2020). Working conditions. Retrieved from http://www.ilo.ch/global/topics/working-conditions/lang--en/index.htm

[ref38] Ismail, H., & Gali, N. (2017). Relationships among performance appraisal satisfaction, work–family conflict and job stress. Journal of Management & Organization, 23(3), 356–372.

[ref39] Kaufman, E., Lovich, D., Bailey, A., Messenböck, R., Schuler, F., & Shroff, A. (2020). Remote work works – where do we go from Here? Retrieved from https://www.bcg.com/publications/2020/remote-work-works-so-where-do-we-go-from-here

[ref40] Koirala, J., & Acharya, S. (2020). Dimensions of human resource management evolved with the outbreak of COVID-19. Available at SSRN 3584092.

[ref41] Kretchmer, H. (2020). How coronavirus has hit employment in G7 economies. Retrieved from https://www.weforum.org/agenda/2020/05/coronavirus-unemployment-jobs-work-impact-g7-pandemic/

[ref42] Leighton, P., & McKeown, T. (2020). Work in challenging and uncertain times: The changing employment relationship. New York: Routledge.

[ref43] Leonardi, P. M. (2020). COVID-19 and the new technologies of organizing: digital exhaust, digital footprints, and artificial intelligence in the wake of remote work. Journal of Management Studies. https://ideas.repec.org/a/bla/jomstd/v58y2021i1p249-253.html.

[ref44] Liu, Y., Lee, J. M., & Lee, C. (2020). The challenges and opportunities of a global health crisis: The management and business implications of COVID-19 from an Asian perspective. Asian Business & Management, 19, 277–297. 10.1057/s41291-020-00119-x PMC7216126 40476954

[ref45] Lund, S., Madgavkar, A., Manyika, J., Smit, S., Ellingrud, K., Meaney, M., & Robinson, O. (2021). The future of work after COVID-19. Retrieved from https://www.mckinsey.com/featured-insights/future-of-work/the-future-of-work-after-covid-19

[ref46] Maclean, J. C., Pichler, S., & Ziebarth, N. R. (2020). Mandated sick pay: Coverage, utilization, and welfare effects. National Bureau of Economic Research,(No. w26832). Retrieved from https://www.nber.org/system/files/working_papers/w26832/w26832.pdf.

[ref47] Major, L. E., & Machin, S. (2020). Covid-19 and social mobility. Centre for Economic Performance, London School of Economics and Political Science, (No.004).

[ref48] Mangan, D. (2020). Covid-19 and labour law in the United Kingdom. European Labour Law Journal, 11(3), 332–346. doi: 10.1177/2031952520934583

[ref49] Martocchio, J. J. (2017). Strategic Compensation: A Human Resource Management Approach. US: Pearson Education.

[ref50] Maurer, R. (2020). Job interviews go virtual in response to COVID-19. Retrieved from https://www.shrm.org/resourcesandtools/hr-topics/talent-acquisition/pages/job-interviews-go-virtual-response-covid-19-coronavirus.aspx

[ref51] Molino, M., Ingusci, E., Signore, F., Manuti, A., Giancaspro, M. L., Russo, V., … Cortese, C. G. (2020). Wellbeing costs of technology use during Covid-19 remote working: an investigation using the Italian translation of the technostress creators scale. Sustainability, 12(15), 5911.

[ref52] Mondy, R. W., & Martocchio, J. J. (2016). Human resource management. USA: Pearson.

[ref53] Navío-Marco, J., Solórzano-García, M., & Palencia-González, F. J. (2019). Human resource management as key pillar of company strategy: Analysis of the line managers’ perception. Journal of Management & Organization, 25(2), 175–188.

[ref54] Ngoc Su, D., Luc Tra, D., Thi Huynh, H. M., Nguyen, H. H. T., & O'Mahony, B. (2021). Enhancing resilience in the Covid-19 crisis: Lessons from human resource management practices in Vietnam. Current Issues in Tourism, 1–17. doi: 10.1080/13683500.2020.1863930

[ref55] Ployhart, R. E. (2006). Staffing in the 21st century: New challenges and strategic opportunities. Journal of Management, 32(6), 868–897.

[ref56] Prasad, K., & Vaidya, R. W. (2020). Association among Covid-19 parameters, occupational stress and employee performance: An empirical study with reference to agricultural research sector in Hyderabad Metro. Sustainable Humanosphere, 16(2), 235–253.

[ref57] Przytuła, S., Strzelec, G., & Krysińska-Kościańska, K. (2020). Re-vision of future trends in human resource management (HRM) after COVID-19. Journal of Intercultural Management, 12(4), 70–90.

[ref58] Qiu, J., Shen, B., Zhao, M., Wang, Z., Xie, B., & Xu, Y. (2020). A nationwide survey of psychological distress among Chinese people in the COVID-19 epidemic: Implications and policy recommendations. General Psychiatry, 33(2), 1–3. doi: 10.1136/gpsych-2020-100213 PMC7061893 32215365

[ref59] Quaedackers, J. S., Stein, R., Bhatt, N., Dogan, H. S., Hoen, L., Nijman, R. J., … Bogaert, G. (2020). Clinical and surgical consequences of the COVID-19 pandemic for patients with pediatric urological problems. Statement of the EAU guidelines panel for paediatric urology, March 30 2020. Journal of Pediatric Urology, 16, 284–287. 32291208 10.1016/j.jpurol.2020.04.007 PMC7144609

[ref60] Regmi, K., & Lwin, C. M. (2020). Impact of social distancing measures for preventing coronavirus disease 2019 [COVID-19]: A systematic review and meta-analysis protocol. medRxiv. doi: 10.1101/2020.06.13.20130294

[ref61] Rothstein, M. A., Parmet, W. E., & Reiss, D. R. (2021). Employer-mandated vaccination for COVID-19. American Public Health Association, ahead of print. Retrieved from https://ajph.aphapublications.org/doi/pdf/10.2105/AJPH.2020.306166 10.2105/AJPH.2020.306166 PMC8101589 33539177

[ref62] Sachs, T. (2020). Covid-19 and labour law in France. European Labour Law Journal, 11(3), 286–291. doi: 10.1177/2031952520934565

[ref63] Safuan, S., & Kurnia, T. (2021). Literature review of pandemic Covid 19 effects on employee compensation. Journal of Business Management Review, 2(1), 57–64.

[ref64] Sagan, A., & Schüller, C. (2020). Covid-19 and labour law in Germany. European Labour Law Journal, 11(3), 292–297. doi: 10.1177/2031952520934566

[ref65] Schneider, D. (2020). Paid sick leave in Washington state: Evidence on employee outcomes, 2016–2018. American Journal of Public Health, 110(4), 499–504. 32078341 10.2105/AJPH.2019.305481 PMC7067115

[ref66] Schuler, R. S. (1992). Strategic human resources management: Linking the people with the strategic needs of the business. Organizational Dynamics, 21(1), 18–32.

[ref67] Sembiring, M. J., Fatihudin, D., Mochklas, M., & Holisin, I. (2020). Banking employee performance during pandemic Covid-19: Remuneration and motivation. Journal of Xi'an University of Architecture & Technology, 12(VII), 64–71.

[ref68] Shaw, W. S., Main, C. J., Findley, P. A., Collie, A., Kristman, V. L., & Gross, D. P.. (2020). Opening the workplace after COVID-19: What lessons can be learned from return-to-work research?. Journal of Occupational Rehabilitation, 30, 299–302. 32562129 10.1007/s10926-020-09908-9 PMC7303572

[ref69] Spurk, D., & Straub, C. (2020). Flexible employment relationships and careers in times of the COVID-19 pandemic. Journal of Vocational Behavior, 103435, 1–4. 10.1016/j.jvb.2020.103435 PMC7204672 32382161

[ref70] Tan, W., Hao, F., McIntyre, R. S., Jiang, L., Jiang, X., Zhang, L., … Luo, X. (2020). Is returning to work during the COVID-19 pandemic stressful? A study on immediate mental health status and psychoneuroimmunity prevention measures of Chinese workforce. Brain, Behavior, and Immunity, 87, 84–92. doi: 10.1016/j.bbi.2020.04.055 PMC7179503 32335200

[ref71] Wang, J., Hutchins, H. M., & Garavan, T. N. (2009). Exploring the strategic role of human resource development in organizational crisis management. Human Resource Development Review, 8(1), 22–53.

[ref72] WHO. (2020a). Critical preparedness, readiness and response actions for COVID-19: Interim guidance, 24 June 2020. Retrieved from https://www.who.int/publications/i/item/critical-preparedness-readiness-and-response-actions-for-covid-19

[ref73] WHO. (2020b). WHO Director-General's opening remarks at the media briefing on COVID-19 – 11 March 2020. Retrieved from https://www.who.int/dg/speeches/detail/who-director-general-s-opening-remarks-at-the-media-briefing-on-covid-19---11-march-2020

[ref74] Wong, E., Ho, K., Wong, S., Cheung, A., & Yeoh, E. (2020). Workplace safety and coronavirus disease (COVID-19) pandemic: survey of employees. Bull World Health Organ. E-pub, 20.

[ref75] World Economic Forum. (2020). COVID-19's workforce impact. Strategic Intelligence. Retrieved from https://intelligence.weforum.org/topics/a1G0X000006O6EHUA0?tab=publications

[ref76] Wright, P. M., & McMahan, G. C. (1992). Theoretical perspectives for strategic human resource management. Journal of Management, 18(2), 295–320.

[ref77] Yu, Z., Wang, G., Goldman, E., Zangerl, B., Xie, N., Cao, Y., … Maida, M. (2021). COVID-19 vaccine: Call for employees in international transportation industries and international travelers as the first priority in global distribution. Open Medicine, 16(1), 134–138. 33521319 10.1515/med-2021-0210 PMC7811367

[ref78] Zhou, X., Snoswell, C. L., Harding, L. E., Bambling, M., Edirippulige, S., Bai, X., & Smith, A. C. (2020). The role of telehealth in reducing the mental health burden from COVID-19. Telemedicine and e-Health, 26(4), 377–379. 32202977 10.1089/tmj.2020.0068

